# Multidisciplinary Healthcare Providers’ Perspectives on Managing Suspected Elder Abuse in the Healthcare Setting

**DOI:** 10.1093/geroni/igaa057.3214

**Published:** 2020-12-16

**Authors:** Lena Makaroun, Gloria Klima, Michele Nichols, Keri Rodriguez, Ann O’Hare, Ann-Marie Rosland

**Affiliations:** 1 University of Pittsburgh School of Medicine, Pittsburgh, Pennsylvania, United States; 2 VA Pittsburgh Healthcare System Center for Health Equity Research and Promotion, Pittsburgh, Pennsylvania, United States; 3 University of Washington School of Medicine, Seattle, Washington, United States

## Abstract

Elder abuse (EA) is common and has devastating health consequences, yet is rarely detected by healthcare professionals. Veterans are at high risk for EA, and the VA has unique resources (e.g., comprehensive social work services) that can help address EA in the healthcare setting. This qualitative study aimed to assess perceived barriers and facilitators to detecting, reporting, intervening on and monitoring EA for VA providers. Providers from two VA facilities were recruited to participate in a one-on-one semi-structured interview. Transcripts of audio-recorded interviews were analyzed using thematic content analysis. Participants (n=22) were 82% female, age 33-64 years, had 4-25 years practicing in VA, and varied in discipline (e.g., nurse, physician, social worker) and practice setting (e.g., emergency department, geriatrics, primary care). For detecting EA, patient and caregiver cognitive impairment were frequently cited barriers, while an interdisciplinary team approach and ability to do home visits were noted facilitators. Common challenges with reporting EA to adult protective services (APS) were perceived lack of APS follow up and discrepancies in VA provider and APS investigator findings. While removing a patient from an unsafe living situation was a frequently cited successful intervention, providers also expressed feeling conflicted when infringing on patient autonomy. Poor communication with APS, patient loss to follow up, and caregiver interference made monitoring EA cases more difficult; intensive case management and in-home services facilitated monitoring. In conclusion, healthcare professionals see interdisciplinary care, in-home care, and better coordination with APS as key facilitators to managing suspected EA in the healthcare setting.

